# Dietary Characteristics Associated With High Defecation Frequency and Constipation in Japanese Adults: A Cross‐Sectional Study

**DOI:** 10.1002/jgh3.70334

**Published:** 2026-01-09

**Authors:** Hirokazu Taniguchi, Miho Ueda, Yukiko Kobayashi, Takatomo Shima

**Affiliations:** ^1^ Faculty of Agriculture, Ryukoku University Shiga Japan; ^2^ Center for Health Promotion, Japanese Red Cross Kyoto Daiichi Hospital Kyoto Japan; ^3^ Division of Applied Life Sciences Graduate School of Life and Environmental Sciences, Kyoto Prefectural University Kyoto Japan

**Keywords:** bowel movement frequency, defecation frequency, dietary behaviors, dietary characteristics, skipping breakfast

## Abstract

**Aims:**

The aim of this study was to evaluate the association between defecation frequency and dietary characteristics in adult men and women to identify factors associated with appropriate defecation frequency.

**Methods:**

We performed a cross‐sectional study of 11,595 participants (*n* = 6142 men and 5453 women) aged 30–79 years using annual health check data collected between 2018 and 2023. Data on dietary characteristics and defecation frequency were collected using a self‐reported questionnaire. Participants were divided according to defecation frequency into high‐ (≥ 3 times/day) and middle‐frequency defecation groups, and a constipation group (≤ every 3 days).

**Results:**

The proportion of high‐frequency defecation was higher in men, whereas constipation was higher in women. For both sexes, high‐frequency defecation was associated with higher BMI, triglyceride, and hepatic enzyme levels, whereas constipation was associated with lower BMI, triglyceride, and hepatic enzyme levels. Multivariable‐adjusted logistic regression analysis indicated that, in men, high‐frequency defecation was associated with eating a snack after dinner. The risk of constipation was negatively associated with frequent consumption of vegetables in both sexes, and fruits in women only. The prevalence of constipation was positively and strongly associated with skipping breakfast every day in both men and women.

**Conclusion:**

This study found a correlation between increased defecation frequency and obesity‐related characteristics, such as elevated BMI, triglycerides, and hepatic enzyme levels, in both men and women. Fiber‐rich food intake may have a preventive effect against constipation, whereas skipping breakfast every day was associated with constipation prevalence in both sexes.

## 1 Introduction

Functional defecation disorders have been identified as a risk factor for non‐communicable diseases in prospective cohort studies. A recent study reported a bidirectional relationship between irritable bowel syndrome and type 2 diabetes in England [1]. Furthermore, chronic constipation was associated with an elevated risk of incident chronic kidney disease in veterans in the United States [2] and of cardiovascular disease (CVD) events and mortality in populations in Japan [3] and the United States [4, 5].

Other prospective cohort studies have reported an association between defecation frequency and CVD. A low frequency of defecation was associated with an increased risk of CVD mortality in Japanese adults [6]. Both high and low frequencies of defecation were associated with an elevated risk of CVD in Chinese adults [7]. On the other hand, no association was found between defecation frequency and CVD in a study of women in the United States; however, high‐frequency defecation was associated with an increased risk of total mortality [8].

Typical defecation frequency ranges widely from three times per week to three times per day [9, 10, 11, 12]. Defecating less than three times per week was classically defined as constipation [9, 10]. A previous cross‐sectional study reported that constipation in Taiwanese adolescents was associated with female sex, not being overweight/obese, and a low intake of fruits and vegetables [13]. Another cross‐sectional study of British adults reported that defecation frequency was lower in women than in men, and higher in individuals with a higher body mass index (BMI), vegetarians, and vegans [14]. These findings suggest that sex, degree of obesity, and a diet consisting of fruits and vegetables are independent factors of defecation frequency regardless of age.

Previous studies reported an association between defecation frequency and dietary behaviors. Defecation is stimulated subsequent to the ingestion of a meal [15, 16]. Previous studies reported an association between skipping breakfast and low defecation frequency in Japanese females [17, 18]. The prevalence of chronic constipation is approximately 10% in Japan [19]. A typical Japanese dietary pattern, characterized by a high consumption of vegetables and soy products, has been associated with a reduced risk of chronic constipation in children and young women [20, 21].

The factors associated with defecation frequency in relation to dietary behaviors and dietary patterns remain unclear, particularly in men and older women. Moreover, the dietary factors associated with high‐frequency defecation remain to be elucidated. Differences in the association between defecation frequency and metabolic parameters in men and women remain unclear. The aim of this study was to evaluate the association of defecation frequency with dietary characteristics and metabolic parameters in both adult males and females.

## 2 Methods

### 2.1 Study Design and Ethical Approval

We conducted a cross‐sectional study using an opt‐out recruitment method at the Japanese Red Cross Society Kyoto Daiichi Hospital in Kyoto City, Japan. Clinical data were taken from annual health check records (Ningen dock), which are voluntary comprehensive health checkups for early disease detection in Japan [22]. All procedures involving human participants were approved by the Ethical Committees of Japanese Red Cross Society Kyoto Daiichi Hospital (approval number: 1500‐1732), Kyoto Prefectural University (approval number: 347), and Ryukoku University (approval number: 2025‐02). This study was conducted according to the guidelines laid down in the Declaration of Helsinki. Opt‐out informed consent was obtained from all participants.

### 2.2 Study Population and Exclusion Criteria

The inclusion criterion for the study was participants who completed the check‐up. Participation in the health check‐up was generally voluntary; however, a subset of participants underwent the examination as part of employer‐mandated periodic screenings. The clinical data system was contracted to a system management company. Data were collected from 12,806 adult participants (6665 men and 6141 women; aged 18–95 years) at their first annual health check between April 2018 and May 2023. During the data collection period, no participants opted out of the study. Exclusion criteria were missing data (*n* = 432); age < 30 and ≥ 80 years (*n* = 50 and 537, respectively); and hepatitis B surface antigen and hepatitis C virus antibody positivity (*n* = 93 and 99, respectively). Finally, we analyzed 11,595 participants (*n* = 6142 men and 5453 women; aged 30–79 years).

### 2.3 Assessment of Defecation Frequency

Data on defecation frequency was collected using a self‐reported questionnaire. Participants reported the number of bowel movements per day or the interval in days between bowel movements. A high frequency of defecation was defined as three times per day or more (*n* = 464, 4.0%). The standard defecation frequency is more than three times per week [9, 10, 11, 12]; therefore, constipation was defined as a defecation frequency of once every 3 days or less, which is consistent with fewer than three defecations per week [9, 10]. Participants were divided into three groups according to defecation frequency: high‐ (≥ 3 times/day) and middle‐frequency (1–2 times/day or every 2 days) defecation groups, and a constipation group (≤ every 3 days). The questionnaire contained items regarding the regular use of laxatives. An additional questionnaire was used to assess symptoms of bloating and the characteristics of stools, including the presence of hard, soft, thin, or bloody stools. The response options were frequent, sometimes, and none.

### 2.4 Measurements

Anthropometric and clinical measurements were performed as previously described [23]. The measurement items were derived from health checkups that have been conducted annually since 2008 as part of health screening by the Ministry of Health, Labor and Welfare in Japan [24, 25]. The objective of the checkups is to diagnose and treat metabolic syndrome early [24, 25]. BMI was calculated as body weight (kg) divided by the square of height (m). Participants were divided into three groups based on their BMI: lean (< 18.5 kg/m^2^), normal weight (18.5–24.9 kg/m^2^), and obese (≥ 25 kg/m^2^). These categories were based on the definition by the Japan Society for the Study of Obesity [26]. Waist circumference (WC) was measured twice in the standing position at the umbilicus. Blood pressure was measured in a sitting position using an upper‐arm blood pressure monitor. Venous blood samples were collected after at least 12 h of fasting, and chemical analysis was performed using standard techniques. Lipid and glucose parameters were measured by triglyceride, total cholesterol, low‐density lipoprotein cholesterol, high‐density lipoprotein (HDL) cholesterol, fasting plasma glucose, and hemoglobin A1c (HbA1c) National Glycohemoglobin Standardization Program values. Aspartate aminotransferase (AST), alanine aminotransferase (ALT), and gamma‐glutamyl transpeptidase (γ‐GTP) were measured as markers of hepatic damage. Estimated glomerular filtration rate was calculated using serum creatinine, age, and sex. C‐reactive protein (CRP) was measured as an indicator of inflammation.

### 2.5 Lifestyle Questionnaires

Lifestyle behaviors were assessed using a self‐administered questionnaire developed by the Health Department of the Ministry of Health, Labour and Welfare in Japan [27]. Smoking habits were obtained through self‐reporting, and participants were classified as never, former, and current smokers. The definition of smoking included both tobacco products, such as cigarettes, and vaping. Alcohol use was evaluated by the amount and type of alcoholic beverages consumed per week and used to estimate mean ethanol intake per week. The quantity of alcohol consumed was assessed using a question on daily ethanol intake, defined in units of 20 g. One unit was defined as 180 mL of sake, 240 mL of wine, 110 mL of *shochu*, 500 mL of beer, 350 mL of canned *chuhai*, or 60 mL of whiskey. The frequency of alcohol consumption was determined on a scale ranging from none to 7 days per week. Habitual alcohol drinking was defined as consumption of more than 20 g/day ethanol for women and 40 g/day ethanol for men. Habitual physical activity was defined as physical activity for more than 1 h per day. Exercise habits were defined as engaging in at least 30 min of exercise twice per week for at least 1 year, excluding physical activity performed for non‐exercise purposes. Habitual sleep duration was assessed using a self‐reported questionnaire, with sleep hours calculated from the reported bedtime and wake‐up time. Employment status was classified as full‐time, part‐time, and not employed. The participants were asked whether or not they were evening shift workers. Stress levels were categorized as extremely stressed, a little stressed, or not much stressed. The participants replied to questionnaires regarding their medical treatment including treatment for hypertension, dyslipidemia, and type 2 diabetes mellitus.

### 2.6 Dietary Questionnaires

We collected data on dietary characteristics, focusing on healthy and unhealthy dietary patterns in Japanese participants [28, 29, 30]. The questionnaire was based on the standard medical questionnaire developed and designed by the Ministry of Health, Labour, and Welfare in Japan, which is widely used in annual health checkups throughout Japan [31]. Participants were asked about their eating habits, such as whether they often consumed seven food and drink items (vegetables, fruits, soybean products, sesame/nuts, sweet buns/bread with fillings, sweets, and soft drinks); four types of food (noodles/rice bowls, stir–/deep‐fried food, simmered/teriyaki food, and eat out/eat ready‐made food); and four dietary behaviors (fast eating, eating a snack after dinner, skipping breakfast every day, and consuming ≥ 30 different food items per day). The dietary behavior of ‘fast eating’ was defined as a self‐awareness of consuming food more rapidly than others. Fast eating is associated with an increased risk of metabolic syndrome and type 2 diabetes in the Japanese working population [32, 33]. The applicability criteria for “often”, “eating a snack after dinner”, and “consume ≥ 30 different food items per day” were set at three times per week or more. Among factors associated with the frequency of three times per week, previous studies have reported an association between metabolic syndrome and the consumption of vegetables [28], and between steatotic liver disease and the type of food consumed [29].

### 2.7 Statistical Analyses

Analysis was performed for men and women separately. SPSS version 29.0 (SPSS Inc.) was used for statistical analyses. Continuous variables were presented as mean ± standard deviation, and categorical variables were expressed as numbers (%). Differences between defecation frequency groups were assessed using the Kruskal–Wallis test (continuous variables) and Chi‐squared test (categorical variables). Logistic regression analysis was performed to identify risk factors for defecation frequency. In order to examine the associations of high‐frequency defecation (≥ 3 times/day) and constipation (≤ every 3 days) with dietary characteristics, multivariable analyses were performed using middle‐frequency defecation (1–2 times/day or every 2 days) as the reference category. The covariates of age, BMI, smoking habits, physical activity habits, exercise habits, employment status, stress level, medical treatment, and laxative use were adjusted. The existence of multicollinearity among covariates was investigated using Spearman's rank correlation coefficient. Significance was set at *p* < 0.05.

In order to address the possibility of false positive results from multiple statistical tests, the *p*‐values for dietary characteristics were controlled using the false discovery rate (FDR) method for the Chi‐squared test and logistic regression analysis. The FDR threshold was set at 5%, using the Benjamini–Hochberg method [34], ensuring that no more than 5% of significant results were classified as false positives. The calculated *q*‐values were presented as *p*‐values adjusted for FDR (pFDR).

## 3 Results

### 3.1 Defecation Frequency

The dataset taken at the first visit consisted of 12,806 participants aged between 18 and 95 years. We excluded 83 responses (0.6%) for missing data. Of the remaining 12,723 participants, 507 (4.0%) exhibited high‐frequency defecation (≥ 3 times/day). High‐frequency defecation was higher in men (6.0%; *n* = 398/6644) than women (1.8%; *n* = 109/6079). The participants exhibited a middle frequency of defecation of 89.2% (*n* = 11,344), which was comparable between men (90.0%; *n* = 5977) and women (88.3%; *n* = 5367). The prevalence of constipation (≤ every 3 days) was 6.9% overall (*n* = 872), which was higher in women (9.9%; *n* = 603) than in men (4.0%; *n* = 269).

For the 11,595 participants aged 30–79 years, the overall frequencies were 4.0% in the high‐ (*n* = 464) and 89.5% in the middle‐frequency defecation groups (*n* = 10,380), and 6.5% in the constipation group (*n* = 751). As shown in Table 1, the proportion of participants in the high‐frequency defecation group was higher among men (6.0%; *n* = 370) than women (1.7%; *n* = 94). Moreover, the prevalence of constipation was higher in women (9.5%; *n* = 520) than men (3.8%; *n* = 231). The defecation frequency in the middle‐frequency defecation group was similar between male and female participants.

### 3.2 General Characteristics According to Differences of Defecation Frequency

Anthropometric characteristics are shown in Table 1. Male participants in the high‐frequency defecation group were younger compared with the other groups. Regarding female participants, the mean age was lower as defecation frequency decreased. In both men and women, a high frequency of defecation was associated with higher BMI, WC, diastolic blood pressure, triglycerides, AST, ALT, and γ‐GTP. In men, high levels of total cholesterol and HDL cholesterol were observed in those with higher defecation frequency. In women, increased systolic blood pressure, fasting glucose, and HbA1c were associated with higher defecation frequency. In men with constipation and women with a high frequency of defecation, serum CRP levels were higher.

**TABLE 1:** Characteristics of participants according to defecation frequency and sex differences.

	Men (*n* = 6142)				Women (*n* = 5453)			
	High (≥ 3 times/day)	Middle (1–2 times/day or every 2 days)	Constipation (≤ every 3 days)	*p*	High (≥ 3 times/day)	Middle (1–2 times/day or every 2 days)	Constipation (≤ every 3 days)	*p*
*n*	370 (6.0)	5541 (90.2)	231 (3.8)		94 (1.7)	4839 (88.7)	520 (9.5)	
Age, years	54.8 ± 12.4	57.6 ± 12.4	56.6 ± 12.5	**< 0.001**	63.9 ± 9.7	57.7 ± 11.8	53.6 ± 12.0	**< 0.001**
30–49 years	134 (7.2)	1649 (88.6)	78 (4.2)	**0.001**	8 (0.5)	1346 (85.4)	223 (14.1)	**< 0.001**
50–64 years	144 (6.6)	1956 (89.7)	80 (3.7)		30 (1.5)	1858 (90.1)	175 (8.5)	
65–79 years	92 (4.4)	1936 (92.1)	73 (3.5)		56 (3.1)	1635 (90.2)	122 (6.7)	
Height, cm	170.0 ± 6.1	170.0 ± 6.4	170.4 ± 6.2	0.827	154.3 ± 6.1	156.9 ± 5.8	157.5 ± 5.6	**< 0.001**
Weight, kg	70.7 ± 13.3	68.2 ± 11.1	66.1 ± 10.4	**< 0.001**	56.3 ± 12.7	53.9 ± 9.4	53.7 ± 9.2	0.264
BMI, kg/m^2^	24.4 ± 4.1	23.5 ± 3.3	22.7 ± 3.0	**< 0.001**	23.6 ± 4.5	21.9 ± 3.6	21.6 ± 3.3	**< 0.001**
< 18.5	13 (5.4)	215 (89.2)	13 (5.4)	**< 0.001**	10 (1.4)	655 (88.5)	75 (10.1)	**0.001**
18.5 < 25.0	224 (5.4)	3753 (90.4)	174 (4.2)		54 (1.4)	3356 (88.8)	371 (9.8)	
≥ 25.0	133 (7.6)	1573 (89.9)	44 (2.5)		30 (3.2)	828 (88.8)	74 (7.9)	
WC, cm	87.5 ± 10.2	85.4 ± 9.0	83.4 ± 8.6	**< 0.001**	84.2 ± 12.7	79.3 ± 9.6	78.0 ± 9.1	**< 0.001**
SBP, mmHg	131.0 ± 15.5	130.9 ± 16.6	128.7 ± 16.6	0.118	133.2 ± 18.2	125.6 ± 18.7	120.3 ± 17.8	**< 0.001**
DBP, mmHg	79.3 ± 11.3	78.6 ± 11.1	76.9 ± 11.3	**0.029**	76.4 ± 10.9	72.9 ± 11.6	70.0 ± 11.4	**< 0.001**
Triglycerides, mg/dL	142.4 ± 141.3	120.1 ± 97.7	104.1 ± 62.8	**< 0.001**	108.5 ± 58.9	87.7 ± 49.4	81.7 ± 45.7	**< 0.001**
Total cholesterol, mg/dL	205.4 ± 34.6	203.2 ± 32.6	195.9 ± 32.7	**0.004**	209.9 ± 31.6	214.1 ± 34.1	209.1 ± 33.3	**0.004**
LDL cholesterol, mg/dL	115.1 ± 32.4	117.5 ± 29.5	116.1 ± 29.4	0.200	113.0 ± 28.7	120.4 ± 30.0	118.0 ± 29.8	**0.023**
HDL cholesterol, mg/dL	63.3 ± 19.5	62.0 ± 16.1	59.1 ± 14.6	**0.020**	75.1 ± 16.3	76.3 ± 17.7	74.7 ± 17.1	0.213
Fasting glucose, mg/dL	108.2 ± 18.0	108.7 ± 20.1	109.1 ± 21.2	0.725	103.6 ± 14.0	100.9 ± 14.0	98.9 ± 13.8	**< 0.001**
HbA1c, %	5.8 ± 0.6	5.8 ± 0.7	6.0 ± 0.8	0.064	5.8 ± 0.4	5.7 ± 0.5	5.7 ± 0.5	**< 0.001**
AST, IU/L	26.7 ± 13.1	23.5 ± 9.8	22.1 ± 10.5	**< 0.001**	23.1 ± 8.0	21.0 ± 8.2	19.6 ± 5.9	**< 0.001**
ALT, IU/L	30.7 ± 23.5	25.2 ± 17.3	22.6 ± 15.3	**< 0.001**	22.4 ± 14.4	17.9 ± 12.2	15.7 ± 9.0	**< 0.001**
γ‐GTP, IU/L	69.4 ± 111.6	44.1 ± 48.0	33.2 ± 40.6	**< 0.001**	29.8 ± 36.1	24.3 ± 25.0	20.7 ± 17.8	**< 0.001**
eGFR, mL/min/1.73m^2^	71.3 ± 14.2	68.5 ± 13.5	69.3 ± 14.4	**< 0.001**	71.1 ± 11.9	70.1 ± 13.3	70.9 ± 13.2	0.160
CRP, mg/dL	0.127 ± 0.395	0.126 ± 0.452	0.140 ± 0.859	**0.011**	0.149 ± 0.314	0.093 ± 0.308	0.081 ± 0.292	**< 0.001**

*Note:* Data are the mean ± standard deviations (continuous variables) or *n* (%) (categorical variables). Significant difference among defecation frequency according to sex difference using the Kruskal–Wallis Test (continuous variables) or Chi‐square test (categorical variables). Boldface indicates significance (*p* < 0.05).

Abbreviations: ALT, alanine aminotransferase; AST, aspartate aminotransferase; BMI, body mass index; CRP, C‐reactive protein; DBP, diastolic blood pressure; eGFR, estimated glomerular filtration rate; HbA1c, glycated hemoglobin A1c; HDL, high‐density lipoprotein; LDL, low‐density lipoprotein; SBP, systolic blood pressure; WC; waist circumference; γ‐GTP, gamma‐glutamyl transpeptidase.

Table 2 shows the general characteristics of the study participants. As defecation frequency increased, male participants showed a higher prevalence of alcohol consumption. A low prevalence of physical activity and exercise habits was observed in both men and women with constipation. The high‐frequency defecation group exhibited a higher percentage of full‐time male workers, whereas the proportion of full‐time female workers was lower. The proportion of participants in the high‐frequency defecation and constipation groups who reported being ‘extremely stressed’ was higher than in the middle‐frequency defecation group. The proportion of men diagnosed with type 2 diabetes was higher in the constipation group. The female participants in the high‐frequency defecation group exhibited higher prevalences of hypertension and dyslipidemia. The use of laxatives was more prevalent among both males and females with constipation. An increase in age was associated with a higher prevalence of laxative use among male and female participants.

**TABLE 2:** Lifestyle, social, stress, and medical characteristics of participants according to defecation frequency and sex differences.

	Men (*n* = 6142)				Women (*n* = 5453)			
	High (≥ 3 times/day)	Middle (1–2 times/day or every 2 days)	Constipation (≤ every 3 days)	*p*	High (≥ 3 times/day)	Middle (1–2 times/day or every 2 days)	Constipation (≤ every 3 days)	*p*
	*n* (%)	*n* (%)	*n* (%)		*n* (%)	*n* (%)	*n* (%)	
Smoking habits								
None	106 (28.6)	1932 (34.9)	89 (38.5)	**< 0.001**	78 (83.0)	3947 (81.6)	413 (79.4)	0.168
Past	174 (47.0)	2609 (47.1)	83 (35.9)		12 (12.8)	668 (13.8)	70 (13.5)	
Current	90 (24.3)	1000 (18.0)	59 (25.5)		4 (4.3)	224 (4.6)	37 (7.1)	
Drinking habits	116 (31.4)	922 (16.6)	22 (9.5)	**< 0.001**	12 (12.8)	499 (10.3)	44 (8.5)	0.292
Physical activity habits	83 (22.4)	1026 (18.5)	27 (11.7)	**0.004**	19 (20.2)	912 (18.8)	72 (13.8)	**0.018**
Exercise habits	138 (37.3)	2129 (38.4)	63 (27.3)	**0.003**	42 (44.7)	1622 (33.5)	112 (21.5)	**< 0.001**
Sleeping hours								
< 6 h	58 (15.7)	648 (11.7)	34 (14.7)	0.082	15 (16.0)	728 (15.0)	100 (19.2)	0.174
≥ 6 to < 9 h	286 (77.3)	4492 (81.1)	176 (76.2)		75 (79.8)	3896 (80.5)	397 (76.3)	
≥ 9 h	26 (7.0)	401 (7.2)	21 (9.1)		4 (4.3)	215 (4.4)	23 (4.4)	
Employment status								
Full‐time	298 (80.5)	3931 (70.9)	168 (72.7)	**0.003**	20 (21.3)	2029 (41.9)	271 (52.1)	**< 0.001**
Part‐time	11 (3.0)	231 (4.2)	8 (3.5)		12 (12.8)	781 (16.1)	100 (19.2)	
None	61 (16.5)	1379 (24.9)	55 (23.8)		62 (66.0)	2029 (41.9)	149 (28.7)	
Evening shift	32 (8.6)	399 (7.2)	22 (9.5)	0.261	2 (2.1)	174 (3.6)	27 (5.2)	0.134
Stress level								
Extremely	83 (22.4)	825 (14.9)	43 (18.6)	**< 0.001**	28 (29.8)	1032 (21.3)	145 (27.9)	**0.001**
Little	188 (50.8)	2888 (52.1)	133 (57.6)		45 (47.9)	2718 (56.2)	282 (54.2)	
Not much	99 (26.8)	1828 (33.0)	55 (23.8)		21 (22.3)	1089 (22.5)	93 (17.9)	
Hypertension	116 (31.4)	1615 (29.1)	64 (27.7)	0.582	40 (42.6)	932 (19.3)	77 (14.8)	**< 0.001**
Dyslipidemia	74 (20.0)	1336 (24.1)	50 (21.6)	0.147	44 (46.8)	1234 (25.5)	95 (18.3)	**< 0.001**
Type 2 diabetes	28 (7.6)	610 (11.0)	40 (17.3)	**0.001**	8 (8.5)	212 (4.4)	26 (5.0)	0.137
Laxative use	22 (5.9)	282 (5.1)	39 (16.9)	**< 0.001**	12 (12.8)	542 (11.2)	136 (26.2)	**< 0.001**
30–49 years old	2 (1.5)	28 (1.7)	3 (3.8)	**< 0.001**	0 (0.0)	109 (8.1)	43 (19.3)	**< 0.001**
50–64 years old	10 (6.9)	67 (3.4)	11 (13.8)		1 (3.3)	178 (9.6)	51 (29.1)	
65–79 years old	10 (10.9)	187 (9.7)	25 (34.2)		11 (19.6)	255 (15.6)	42 (34.4)	

*Note:* Data are *n* (%). Significant difference among defecation frequency according to sex difference: Chi‐square test. Boldface indicates significance (*p* < 0.05).

Bloating symptoms and stool characteristics are shown in Table S1. A high frequency of defecation was associated with symptoms of bloating among the males. On the other hand, symptoms of bloating were associated with constipation among the females. The proportion of hard stools was higher in the constipation group, both for males and females. A high frequency of defecation was associated with higher percentages of soft and thin stools among both sexes.

### 3.3 Dietary Characteristics According to Differences of Defecation Frequency

Table 3 shows the results of Chi‐square test for dietary characteristics. Male participants with a high defecation frequency were more likely to often eat soybean products, sweet buns/bread with fillings, noodles/rice bowls, stir−/deep‐fried food, simmered/teriyaki food, out/ready‐made food, and a snack after dinner. Among male participants with constipation, “often eat vegetables” and “soybean products” were lower, whereas “often eat simmered/teriyaki food”, “often eat out/ready‐made food”, and the prevalence of skipping breakfast every day were higher.

**TABLE 3:** Dietary characteristics of participants according to defecation frequency and sex differences.

	Men (*n* = 6142)				Women (*n* = 5453)			
	High (≥ 3 times/day)	Middle (1–2 times/day or every 2 days)	Constipation (≤ every 3 days)	*pFDR*	High (≥ 3 times/day)	Middle (1–2 times/day or every 2 days)	Constipation (≤ every 3 days)	*pFDR*
	*n* (%)	*n* (%)	*n* (%)		*n* (%)	*n* (%)	*n* (%)	
Food preferences								
Vegetables	215 (58.1)	3182 (57.4)	107 (46.3)	**0.011**	67 (71.3)	3476 (71.8)	300 (57.7)	**< 0.001**
Fruits	183 (49.5)	3081 (55.6)	114 (49.4)	**0.023**	69 (73.4)	3408 (70.4)	296 (56.9)	**< 0.001**
Soybean products	171 (46.2)	2241 (40.4)	76 (32.9)	**0.011**	51 (54.3)	2532 (52.3)	233 (44.8)	**0.006**
Sesame/nuts	72 (19.5)	1049 (18.9)	32 (13.9)	0.166	32 (34.0)	1465 (30.3)	114 (21.9)	**< 0.001**
Sweet buns/bread with fillings	75 (20.3)	780 (14.1)	35 (15.2)	**0.011**	9 (9.6)	296 (6.1)	29 (5.6)	0.354
Sweets	225 (60.8)	3492 (63.0)	149 (64.5)	0.614	73 (77.7)	4033 (83.3)	445 (85.6)	0.168
Soft drinks	99 (26.8)	1346 (24.3)	68 (29.4)	0.160	26 (27.7)	1184 (24.5)	158 (30.4)	**0.015**
Food styles								
Noodles/rice bowls	170 (45.9)	2092 (37.8)	84 (36.4)	**0.011**	19 (20.2)	1039 (21.5)	126 (24.2)	0.354
Stir−/deep‐fried food	185 (50.0)	2211 (39.9)	81 (35.1)	**< 0.001**	31 (33.0)	1535 (31.7)	178 (34.2)	0.496
Simmered/teriyaki food	88 (23.8)	1016 (18.3)	55 (23.8)	**0.011**	37 (39.4)	1513 (31.3)	116 (22.3)	**< 0.001**
Eating out/ready‐made food	128 (34.6)	1543 (27.8)	74 (32.0)	**0.015**	25 (26.6)	983 (20.3)	157 (30.2)	**< 0.001**
Dietary behaviors								
Fast eating	227 (61.4)	3187 (57.5)	125 (54.1)	0.206	56 (59.6)	2201 (45.5)	263 (50.6)	**0.005**
Eating a snack after dinner	109 (29.5)	1184 (21.4)	52 (22.5)	**0.005**	28 (29.8)	1253 (25.9)	181 (34.8)	**< 0.001**
Skipping breakfast every day	39 (10.5)	439 (7.9)	40 (17.3)	**< 0.001**	3 (3.2)	217 (4.5)	53 (10.2)	**< 0.001**
Consume ≥ 30 foods per day	32 (8.6)	379 (6.8)	9 (3.9)	0.109	19 (20.2)	687 (14.2)	40 (7.7)	**< 0.001**

*Note:* Data are *n* (%). Significant difference among defecation frequency according to sex difference: Chi‐square test. Boldface indicates significance (*pFDR* < 0.05).

Abbreviation: *pFDR*, adjusted *p*‐value by false discovery rate.

For female participants, a higher percentage of “often eat simmered/teriyaki food”, “fast eating”, and “consume ≥ 30 foods per day” was observed in the high‐frequency defecation group. The prevalence of constipation was associated with lower percentages of “often eat vegetables”, “fruits”, “soybean products”, “sesame/nuts”, “simmered/teriyaki food”, and “consume ≥ 30 foods per day”. Female participants with constipation were more likely to “often consume soft drinks”, “often eat out/ready‐made food”, “eating a snack after dinner”, and “skipping breakfast every day”.

### 3.4 Associations Between High‐Frequency Defecation (≥ 3 Times/Day) and Dietary Characteristics

Logistic regression analysis was performed to evaluate the association between high‐frequency defecation and dietary characteristics (Table 4). The age‐ and BMI‐adjusted model for men showed that “often eat soybean products”, “sweet buns/bread with fillings”, “stir−/deep‐fried food”, “simmered/teriyaki food”, and “eating a snack after dinner” were associated with a higher chance of high‐frequency defecation. In a multivariable‐adjusted model, the association between high‐frequency defecation and dietary characteristics remained significant in male participants, except for the variable “often eat stir‐/deep‐fried food”.

**TABLE 4:** Association between dietary characteristics and high defecation frequency (≥ 3 times/day) from multivariable‐adjusted logistic regression analyses in men and women.

	Men (*n* = 6142)						Women (*n* = 5453)					
	Age and BMI adjusted			Multivariable adjusted^a^			Age and BMI adjusted			Multivariable adjusted^a^		
	OR	95% CI	*pFDR*	OR	95% CI	*pFDR*	OR	95% CI	*pFDR*	OR	95% CI	*pFDR*
Food preferences												
Vegetables	1.11	(0.89–1.37)	0.520	1.17	(0.94–1.46)	0.337	0.84	(0.53–1.33)	0.687	0.80	(0.50–1.27)	0.588
Fruits	0.92	(0.73–1.15)	0.520	1.10	(0.87–1.39)	0.605	0.75	(0.45–1.24)	0.615	0.73	(0.43–1.22)	0.588
Soybean products	1.35	(1.09–1.67)	**0.030**	1.40	(1.13–1.74)	**0.010**	0.94	(0.62–1.43)	0.922	0.92	(0.60–1.40)	0.850
Sesame/nuts	1.10	(0.84–1.44)	0.520	1.09	(0.83–1.43)	0.649	1.04	(0.67–1.61)	0.922	1.02	(0.66–1.59)	0.962
Sweet buns/bread with fillings	1.41	(1.08–1.85)	**0.036**	1.43	(1.09–1.88)	**0.041**	1.63	(0.81–3.30)	0.513	1.54	(0.75–3.14)	0.588
Sweets	0.93	(0.74–1.15)	0.520	1.06	(0.85–1.33)	0.696	0.68	(0.42–1.12)	0.488	0.71	(0.43–1.18)	0.588
Soft drinks	1.10	(0.86–1.39)	0.520	1.18	(0.92–1.50)	0.337	1.26	(0.79–2.00)	0.615	1.29	(0.81–2.05)	0.588
Food styles												
Noodles/rice bowls	1.28	(1.03–1.58)	0.068	1.15	(0.93–1.44)	0.337	0.90	(0.54–1.50)	0.922	0.85	(0.51–1.43)	0.762
Stir−/deep‐fried food	1.33	(1.07–1.65)	**0.036**	1.26	(1.01–1.57)	0.095	1.19	(0.76–1.85)	0.687	1.14	(0.73–1.79)	0.762
Simmered/teriyaki food	1.58	(1.22–2.04)	**< 0.01**	1.60	(1.24–2.08)	**< 0.01**	1.03	(0.67–1.59)	0.922	1.01	(0.65–1.57)	0.962
Eating out/ready‐made food	1.19	(0.95–1.49)	0.264	1.10	(0.88–1.39)	0.602	1.68	(1.04–2.70)	0.255	1.71	(1.05–2.78)	0.240
Dietary behaviors												
Fast eating	1.04	(0.84–1.30)	0.703	1.01	(0.81–1.26)	0.929	1.65	(1.08–2.52)	0.255	1.64	(1.07–2.51)	0.240
Eating a snack after dinner	1.42	(1.12–1.79)	**0.030**	1.50	(1.18–1.90)	**< 0.01**	1.47	(0.93–2.33)	0.488	1.52	(0.95–2.42)	0.400
Skipping breakfast every day	1.15	(0.81–1.64)	0.520	0.97	(0.68–1.41)	0.929	1.06	(0.33–3.43)	0.922	0.94	(0.28–3.12)	0.962
Consume ≥ 30 foods per day	1.49	(1.02–2.19)	0.088	1.57	(1.06–2.31)	0.069	1.30	(0.78–2.19)	0.615	1.28	(0.76–2.16)	0.588

*Note:* The results of a multivariable logistic regression are shown. The reference was middle defecation frequency (1–2 times/day or every 2 days). Bold indicates significance at *pFDR* < 0.05.

Abbreviations: BMI, body mass index; CI, confidence interval; OR, odds ratio; *pFDR*, adjusted *p*‐value by false discovery rate.

^a^ Adjusted for age, BMI, smoking habits, drinking habits, physical activity habits, exercise habits, employment status, stress level, medical treatment, and laxative use.

Regarding the female participants, pFDR showed no significant association between dietary characteristics and high‐frequency defecation. Table S2 shows the unadjusted *p*‐values for the multivariable‐adjusted model.

### 3.5 Associations Between Constipation and Dietary Characteristics

Table 5 shows associations between the prevalence of constipation and dietary characteristics. Results from the two logistic models in male participants indicated that “often eat vegetables” was significantly associated with a lower risk of constipation. Additionally, among dietary characteristics, “skipping breakfast every day” was identified as a strong risk factor for constipation in men.

**TABLE 5:** Association between dietary characteristics and constipation (≤ every 3 days) from multivariable‐adjusted logistic regression analyses in men and women.

	Men (*n* = 6142)						Women (*n* = 5453)					
	Age and BMI adjusted			Multivariable adjusted^a^			Age and BMI adjusted			Multivariable adjusted^a^		
	OR	95% CI	*pFDR*	OR	95% CI	*pFDR*	OR	95% CI	*pFDR*	OR	95% CI	*pFDR*
Food preferences												
Vegetables	0.65	(0.50–0.85)	**0.008**	0.68	(0.52–0.90)	**0.045**	0.59	(0.49–0.71)	**< 0.01**	0.61	(0.50–0.74)	**< 0.01**
Fruits	0.78	(0.59–1.04)	0.169	0.81	(0.60–1.08)	0.283	0.68	(0.56–0.82)	**< 0.01**	0.70	(0.57–0.86)	**< 0.01**
Soybean products	0.72	(0.54–0.95)	0.082	0.74	(0.56–0.99)	0.154	0.81	(0.67–0.97)	**0.030**	0.85	(0.70–1.02)	0.128
Sesame/nuts	0.70	(0.48–1.02)	0.169	0.74	(0.51–1.09)	0.283	0.73	(0.58–0.90)	**0.009**	0.75	(0.60–0.94)	**0.030**
Sweet buns/bread with fillings	1.10	(0.76–1.59)	0.699	1.07	(0.73–1.56)	0.792	0.87	(0.58–1.29)	0.513	0.81	(0.54–1.22)	0.361
Sweets	1.09	(0.82–1.43)	0.699	1.00	(0.76–1.33)	0.981	1.18	(0.91–1.53)	0.255	1.08	(0.83–1.41)	0.620
Soft drinks	1.31	(0.98–1.75)	0.169	1.20	(0.89–1.61)	0.369	1.27	(1.04–1.55)	**0.030**	1.17	(0.95–1.43)	0.194
Food styles												
Noodles/rice bowls	0.97	(0.74–1.28)	0.844	0.95	(0.71–1.25)	0.792	1.14	(0.92–1.41)	0.272	1.13	(0.90–1.40)	0.360
Stir−/deep‐fried food	0.83	(0.63–1.10)	0.305	0.80	(0.60–1.07)	0.283	1.02	(0.84–1.24)	0.872	1.02	(0.84–1.25)	0.831
Simmered/teriyaki food	1.50	(1.09–2.06)	0.070	1.52	(1.10–2.11)	0.055	0.76	(0.61–0.95)	**0.030**	0.77	(0.61–0.97)	**0.043**
Eating out/ready‐made food	1.27	(0.95–1.70)	0.172	1.19	(0.89–1.60)	0.369	1.52	(1.24–1.87)	**< 0.01**	1.45	(1.18–1.79)	**< 0.01**
Dietary behaviors												
Fast eating	0.94	(0.71–1.23)	0.699	0.91	(0.69–1.19)	0.667	1.24	(1.03–1.49)	**0.030**	1.21	(1.01–1.46)	0.073
Eating a snack after dinner	1.08	(0.78–1.48)	0.699	0.94	(0.68–1.30)	0.792	1.37	(1.13–1.67)	**0.003**	1.28	(1.05–1.56)	**0.036**
Skipping breakfast every day	2.46	(1.70–3.57)	**< 0.01**	2.36	(1.60–3.49)	**< 0.01**	2.01	(1.46–2.77)	**< 0.01**	1.99	(1.43–2.78)	**< 0.01**
Consume ≥ 30 foods per day	0.55	(0.28–1.08)	0.169	0.56	(0.28–1.12)	0.283	0.59	(0.42–0.82)	**0.005**	0.62	(0.44–0.88)	**0.021**

*Note:* The results of a multivariable logistic regression are shown. The reference was middle defecation frequency (1–2 times/day or every 2 days). Bold indicates significance at *pFDR* < 0.05.

Abbreviations: BMI, body mass index; CI, confidence interval; OR, odds ratio; pFDR, adjusted *p*‐value by false discovery rate.

^a^ Adjusted for age, BMI, smoking habits, drinking habits, physical activity habits, exercise habits, employment status, stress level, medical treatment, and laxative use.

In the age‐ and BMI‐adjusted logistic model, the prevalence of constipation among female participants was negatively associated with “often eat soybean products” and positively associated with “often consume soft drinks” and “fast eating”. Following adjustment for age and BMI, multivariable‐adjusted models revealed that dietary characteristics, including “often eat vegetables”, “fruits”, “sesame/nuts”, “simmered/teriyaki food”, and “consume ≥ 30 foods per day” were associated with a lower risk of constipation among female participants. For the females, “skipping breakfast every day” was identified as a strong risk factor of constipation among dietary characteristics. In addition, “often eat out/ready‐made food” and “eating a snack after dinner” were also risk factors for constipation among women.

## 4 Discussion

This cross‐sectional study evaluated the association between defecation frequency and dietary characteristics in male and female adults. For male participants, a significant association was identified between high‐frequency defecation and various dietary characteristics. “Skipping breakfast every day” was identified as a strong and common risk factor for constipation in both male and female participants. For females, other dietary characteristics, including “often eat out/ready‐made food” and “eating a snack after dinner”, were identified as risk factors for constipation. These findings suggest that the determinants of defecation are not solely influenced by food consumption but also by dietary characteristics.

Both male and female participants with high defecation frequency exhibited higher BMI, WC, triglyceride, and hepatic enzyme levels. Among male participants, a positive association was found between high defecation frequency and the dietary characteristics of often eating soybean products, sweet buns/bread with fillings, simmered/teriyaki food, and eating a snack after dinner. These findings suggest that excess energy intake and/or storage are associated with frequent defecation. Moreover, we found a higher frequency of defecation in men who exhibited a higher frequency of alcohol consumption. These findings are consistent with previous studies, which reported a negative association between daily alcohol consumption and the prevalence of constipation [35, 36]. On the other hand, a relationship between drinking habits and defecation frequency was not observed among female participants. This sex discrepancy may be attributable to the lower prevalence of alcohol consumption among women than men. The present study found elevated levels of stress in the high‐frequency defecation group in both sexes. Stress is a prevalent risk factor for the consumption of unhealthy foods and the development of obesity [37]; thus, high defecation frequency may be associated with stress‐induced excess food consumption. Furthermore, the female participants in the higher defecation group exhibited a higher prevalence of hypertension and dyslipidemia, which may be attributable to older age and high BMI [38, 39].

Reported risk factors for chronic constipation include female sex [40, 41], increasing age [40, 41], and low physical activity [42, 43]. Regarding sex, the present study observed a more than twofold higher prevalence of constipation in women compared with men. Regarding low physical activity, the constipation group in both sexes displayed a lower level of physical activity and exercise habits. Regarding age, the mean age of the male constipation group was not notably lower, whereas the female constipation group was younger compared with the other groups. In addition, constipation in both males and females was found to be associated with lower BMI, WC, triglyceride, and hepatic enzyme levels. Our findings suggest an association between lower food intake and constipation in Japanese individuals, particularly among women, except for older women. The present study found that higher levels of stress were observed among male and female participants with constipation. The existence of a relationship between perceived stress and unhealthy diets, characterized by high‐fat, low‐fruit, and low‐vegetable intakes, has been previously reported [44, 45]. Our findings suggest an association between stress levels and unhealthy diet in the constipation group. We found a higher prevalence of type 2 diabetes in the male participants with constipation in this study. This supports the findings of a previous study that reported that patients with type 2 diabetes exhibited a higher prevalence of constipation [46].

The present study found that skipping breakfast every day is a significant risk factor for constipation among both male and female adults. Previous Japanese studies reported an association between constipation and skipping breakfast in schoolchildren [47] and adolescents [48]. Moreover, skipping breakfast was associated with a lower frequency of defecation in female college students [17] and working women [18] in Japan. These studies are consistent with the associations found in the present study among both male and female adults. Ingestion of food is associated with an increase in peristaltic activity [49, 50]. Two one‐arm studies with a small sample size reported that skipping breakfast in young women resulted in a reduction in gastric motility [51], and that a nutrition education program to promote breakfast consumption increased gastric motility among high school students [52]. These studies suggest that skipping breakfast reduces intestinal motility, thereby increasing the risk of constipation. Previous studies reported that skipping breakfast was associated with an increased risk of depressive disorder [53], and constipation was prospectively associated with an elevated risk of depression [54]. Although the present study did not investigate depression, constipation induced by skipping breakfast may be a contributing factor to depressive disorder. Further research is required to elucidate the beneficial effects of a daily breakfast on mental health outcomes through the prevention of constipation.

A recent meta‐analysis of randomized controlled trials reported that fiber supplementation exerts a beneficial effect on chronic constipation [55]. Similarly, the present study found that the risk of constipation was lower in participants who often consumed fiber‐rich foods such as vegetables, fruits, and sesame/nuts. The relationship between lower constipation risk and “consume ≥ 30 foods per day” in female participants may also be associated with consumption of foods containing fiber. The present study demonstrated a higher percentage of fiber‐rich food consumption among female participants. In contrast, male participants exhibited a preference for high‐energy food styles, including noodles/rice bowls and stir–/deep‐fried food. The inconsistent associations between specific food items and constipation risk across sexes may be attributed to sex‐related differences in food preferences among Japanese adults. In the Japanese diet, simmered food is typically cooked using fiber‐rich root vegetables, which may explain the negative association between “often eat simmered/teriyaki food” and constipation risk among females. The risk of constipation in females was increased by unfavorable dietary characteristics, including often eating out/ready‐made food and eating a snack after dinner. Although the underlying reason remains unclear, these dietary characteristics may be associated with an inactive lifestyle, which is a recognized risk factor for constipation [42, 43].

This study has several limitations. We used hospital data from annual health checks, a cross‐sectional design, and a non‐quantitative questionnaire. Thus, there was selection bias, and a causal relationship was not confirmed. Data collection was carried out using a questionnaire; therefore, defecation frequency may be underreported. Because constipation was defined by the frequency of defecation, the relationship between dietary characteristics and chronic constipation, as diagnosed by clinical criteria, remains to be elucidated. The beneficial effects of prebiotic compounds in fruits and vegetables on constipation were reported in a previous study [56]; however, the present study did not differentiate between prebiotic foods and other foods. A number of studies have previously reported an association between low fluid intake and constipation [57, 58]; however, the present study did not evaluate total fluid consumption.

## 5 Conclusions

This cross‐sectional study found a high frequency of defecation to be associated with obese status in participants of both sexes and related to frequent food consumption in males. Multivariable adjusted logistic regression analysis revealed a preventative effect of fiber‐rich food consumption on constipation, and an association between skipping breakfast every day and constipation prevalence in both male and female participants. The findings of this study suggest that healthy dietary choices are important for appropriate defecation frequency in adult individuals.

## Funding

The authors have nothing to report.

## Ethics Statement

All procedures involving human participants were approved by the Ethical Committees of Japanese Red Cross Society Kyoto Daiichi Hospital (approval number: 1500‐1732), Kyoto Prefectural University (approval number: 347), and Ryukoku University (approval number: 2025‐02). This study was conducted according to the guidelines laid down in the Declaration of Helsinki.

## Consent

Opt‐out informed consent was obtained from all participants.

## Conflicts of Interest

The authors declare no conflicts of interest.

## Supporting information


**Table S1:** Bloating and stool characteristics of participants according to defecation frequency and sex differences.
**Table S2:** Association of dietary characteristics with high defecation frequency (≥ 3 times/day) and constipation (≤ every 3 days) from multivariable‐adjusted logistic regression analyses in men and women.

## Data Availability

The data that support the findings of this study are available from the corresponding author upon reasonable request.
