# Supplementary material for: Dietary Characteristics Associated With High Defecation Frequency and Constipation in Japanese Adults: A Cross‐Sectional Study
Source: JGH Open. 2026 Jan 9;10(1):e70334. doi: 10.1002/jgh3.70334 (PMC12788986; doi:10.1002/jgh3.70334)
Supplement: Supplementary file 1 — Table S1: Bloating and stool characteristics of participants according to defecation frequency and sex differences. Table S2: Association of dietary characteristics with high defecation frequency (≥ 3 times/day) and constipation (≤ every 3 days) from multivariable‐adjusted logistic regression analyses in men and women. [file JGH3-10-e70334-s001.docx]

| Table S1. Bloating and stool characteristics of participants according to defecation frequency and sex differences. | | | | | | | | | | | | |  |  |  |
| --- | --- | --- | --- | --- | --- | --- | --- | --- | --- | --- | --- | --- | --- | --- | --- |
|  | | Men (n = 6,142) | | | | | |  | Women (n = 5,453) | | | | | |  |
|  |  | High  (≥ 3 times/day) | | Middle  (1–2 times/day or every 2 days) | | Constipation  (≤ every 3 days) | | *p* | High  (≥ 3 times/day) | | Middle  (1–2 times/day or every 2 days) | | Constipation  (≤ every 3 days) | | *p* |
|  |  | n | (%) | n | (%) | n | (%) |  | n | (%) | n | (%) | n | (%) |  |
| Bloating | Frequent | 23 | (6.2) | 173 | (3.1) | 11 | (4.8) | **0.001** | 7 | (7.4) | 219 | (4.5) | 57 | (11.0) | **< 0.001** |
|  | Sometimes | 90 | (24.3) | 1108 | (20.0) | 51 | (22.1) |  | 29 | (30.9) | 1224 | (25.3) | 181 | (34.8) |  |
|  | None | 257 | (69.5) | 4260 | (76.9) | 169 | (73.2) |  | 58 | (61.7) | 3396 | (70.2) | 282 | (54.2) |  |
| Hard stool | Frequent | 13 | (3.5) | 173 | (3.1) | 66 | (28.6) | **< 0.001** | 7 | (7.4) | 257 | (5.3) | 177 | (34.0) | **< 0.001** |
|  | Sometimes | 110 | (29.7) | 1995 | (36.0) | 98 | (42.4) |  | 23 | (24.5) | 1876 | (38.8) | 227 | (43.7) |  |
|  | None | 247 | (66.8) | 3373 | (60.9) | 67 | (29.0) |  | 64 | (68.1) | 2706 | (55.9) | 116 | (22.3) |  |
| Soft stool | Frequent | 130 | (35.1) | 670 | (12.1) | 28 | (12.1) | **< 0.001** | 23 | (24.5) | 393 | (8.1) | 40 | (7.7) | **< 0.001** |
|  | Sometimes | 154 | (41.6) | 2911 | (52.5) | 118 | (51.1) |  | 40 | (42.6) | 2294 | (47.4) | 218 | (41.9) |  |
|  | None | 86 | (23.2) | 1960 | (35.4) | 85 | (36.8) |  | 31 | (33.0) | 2152 | (44.5) | 262 | (50.4) |  |
| Thin stool | Frequent | 47 | (12.7) | 304 | (5.5) | 21 | (9.1) | **< 0.001** | 18 | (19.1) | 217 | (4.5) | 31 | (6.0) | **< 0.001** |
|  | Sometimes | 158 | (42.7) | 1956 | (35.3) | 73 | (31.6) |  | 31 | (33.0) | 1577 | (32.6) | 171 | (32.9) |  |
|  | None | 165 | (44.6) | 3281 | (59.2) | 137 | (59.3) |  | 45 | (47.9) | 3045 | (62.9) | 318 | (61.2) |  |
| Bloody stool | Frequent | 5 | (1.4) | 10 | (0.2) | 1 | (0.4) | **< 0.001** | 0 | (0.0) | 5 | (0.1) | 0 | (0.0) | **0.023** |
|  | Sometimes | 25 | (6.8) | 253 | (4.6) | 20 | (8.7) |  | 2 | (2.1) | 97 | (2.0) | 22 | (4.2) |  |
|  | None | 340 | (91.9) | 5278 | (95.3) | 210 | (90.9) |  | 92 | (97.9) | 4737 | (97.9) | 498 | (95.8) |  |
| Data are n (%). Significant difference among defecation frequency according to sex difference: Chi-square test. Boldface indicates significance (p < 0.05). | | | | | | | | | | | | | | | |

| Table S2. Association of dietary characteristics with high defecation frequency (≥ 3 times/day) and constipation (≤ every 3 days) from multivariable-adjusted logistic regression analyses in men and women. | | | | | | | | | | | | | | | | |
| --- | --- | --- | --- | --- | --- | --- | --- | --- | --- | --- | --- | --- | --- | --- | --- | --- |
|  |  | High (≥ 3 times/day), multivariable adjusted ^†^ | | | | | | |  | Constipation (≤ every 3 days), multivariable adjusted ^†^ | | | | | | |
|  |  | Men (n = 6,142) | | |  | Women (n = 5,453) | | |  | Men (n = 6,142) | | |  | Women (n = 5,453) | | |
|  |  | OR | 95%CI | *p* |  | OR | 95%CI | *p* |  | OR | 95%CI | *p* |  | OR | 95%CI | *p* |
| Food preferences | |  |  |  |  |  |  |  |  |  |  |  |  |  |  |  |
|  | Vegetables | 1.17 | (0.94–1.46) | 0.159 |  | 0.80 | (0.50–1.27) | 0.345 |  | 0.68 | (0.52–0.90) | **0.006** |  | 0.61 | (0.50–0.74) | **<0.001** |
|  | Fruits | 1.10 | (0.87–1.39) | 0.444 |  | 0.73 | (0.43–1.22) | 0.227 |  | 0.81 | (0.60–1.08) | 0.151 |  | 0.70 | (0.57–0.86) | **<0.001** |
|  | Soybean products | 1.40 | (1.13–1.74) | **0.002** |  | 0.92 | (0.60–1.40) | 0.680 |  | 0.74 | (0.56–0.99) | **0.041** |  | 0.85 | (0.70–1.02) | 0.085 |
|  | Sesame/nuts | 1.09 | (0.83–1.43) | 0.519 |  | 1.02 | (0.66–1.59) | 0.926 |  | 0.74 | (0.51–1.09) | 0.132 |  | 0.75 | (0.60–0.94) | **0.012** |
|  | Sweet buns/bread with fillings | 1.43 | (1.09–1.88) | **0.011** |  | 1.54 | (0.75–3.14) | 0.238 |  | 1.07 | (0.73–1.56) | 0.739 |  | 0.81 | (0.54–1.22) | 0.313 |
|  | Sweets | 1.06 | (0.85–1.33) | 0.603 |  | 0.71 | (0.43–1.18) | 0.181 |  | 1.00 | (0.76–1.33) | 0.981 |  | 1.08 | (0.83–1.41) | 0.579 |
|  | Soft drinks | 1.18 | (0.92–1.50) | 0.194 |  | 1.29 | (0.81–2.05) | 0.293 |  | 1.20 | (0.89–1.61) | 0.229 |  | 1.17 | (0.95–1.43) | 0.142 |
| Food styles | |  |  |  |  |  |  |  |  |  |  |  |  |  |  |  |
|  | Noodles/rice bowls | 1.15 | (0.93–1.44) | 0.202 |  | 0.85 | (0.51–1.43) | 0.547 |  | 0.95 | (0.71–1.25) | 0.693 |  | 1.13 | (0.90–1.40) | 0.288 |
|  | Stir-/deep-fried food | 1.26 | (1.01–1.57) | **0.038** |  | 1.14 | (0.73–1.79) | 0.559 |  | 0.80 | (0.60–1.07) | 0.134 |  | 1.02 | (0.84–1.25) | 0.831 |
|  | Simmered/teriyaki food | 1.60 | (1.24–2.08) | **<0.001** |  | 1.01 | (0.65–1.57) | 0.962 |  | 1.52 | (1.10–2.11) | **0.011** |  | 0.77 | (0.61–0.97) | **0.023** |
|  | Eating out/ready-made food | 1.10 | (0.88–1.39) | 0.401 |  | 1.71 | (1.05–2.78) | **0.032** |  | 1.19 | (0.89–1.60) | 0.246 |  | 1.45 | (1.18–1.79) | **<0.001** |
| Dietary behaviors | |  |  |  |  |  |  |  |  |  |  |  |  |  |  |  |
|  | Fast eating | 1.01 | (0.81–1.26) | 0.929 |  | 1.64 | (1.07–2.51) | **0.022** |  | 0.91 | (0.69–1.19) | 0.489 |  | 1.21 | (1.01–1.46) | **0.044** |
|  | Eating a snack after dinner | 1.50 | (1.18–1.90) | **0.001** |  | 1.52 | (0.95–2.42) | 0.080 |  | 0.94 | (0.68–1.30) | 0.713 |  | 1.28 | (1.05–1.56) | **0.017** |
|  | Skipping breakfast every day | 0.97 | (0.68–1.41) | 0.890 |  | 0.94 | (0.28–3.12) | 0.922 |  | 2.36 | (1.60–3.49) | **<0.001** |  | 1.99 | (1.43–2.78) | **<0.001** |
|  | Consume ≥ 30 foods per day | 1.57 | (1.06–2.31) | **0.023** |  | 1.28 | (0.76–2.16) | 0.353 |  | 0.56 | (0.28–1.12) | 0.102 |  | 0.62 | (0.44–0.88) | **0.007** |
| † Adjusted for age, BMI, smoking habits, drinking habits, physical activity habits, exercise habits, employment status, stress level, medical treatment, and laxative use. The results of a multivariable logistic regression are shown. The reference was middle defecation frequency (1–2 times/day or every 2 days). Bold indicates significance at p < 0.05.  BMI, body mass index; CI, confidence interval; OR, odds ratio. | | | | | | | | | | | | | | | | |
